# Protein profiling of ovarian cancers by immunohistochemistry to identify potential target pathways

**DOI:** 10.1186/2053-6844-1-4

**Published:** 2014-09-30

**Authors:** Cassandra D Foss, Heather J Dalton, Bradley J Monk, Dana M Chase, John H Farley

**Affiliations:** Division of Gynecologic Oncology, Department of Obstetrics and Gynecology, University of Arizona Cancer Center, 500 W. Thomas Road, Suite 600, Phoenix, AZ 85013 USA; Creighton University School of Medicine at Dignity Health St. Joseph's Hospital and Medical Center, 500 W. Thomas Road, Suite 600, Phoenix, AZ 85013 USA; Department of Gynecologic Oncology and Reproductive Medicine, The University of Texas MD Anderson Cancer Center, Houston, TX USA

**Keywords:** Protein profiling, Ovarian cancer, Immunohistochemistry

## Abstract

**Background:**

To determine the protein expression profile (PEP) of primary and recurrent ovarian cancer patients in order to predict therapeutic targets for chemotherapy.

**Methods:**

Tissue samples were submitted for PEP in two formats, including formalin-fixed paraffin-embedded tissue for immunohistochemistry (IHC) and fresh frozen tissue for oligonucleotide microarray (MA) gene expression assays. Specimens were analyzed for 18 protein markers and 88 MA genes. A series of Generalized Linear Models (GLM) was used to predict the proportion of positive results by histology for each biomarker.

**Results:**

Four hundred and twenty-eight specimens were analyzed for IHC and 67 specimens for MA analysis. The majority of specimens, 82%, were serous histology and 35.3% of specimens were poorly differentiated. Sixty percent of specimens were advanced stage, 62% were from a primary diagnosis, and 53% were obtained from a metastatic site. BCRP, ER, MGMT, and RRM1 proteins were overexpressed in 85%, 47%, 93%, and 47% of serous carcinomas, respectively. The MGMT and RRM1 biomarkers were significantly overexpressed in serous (p < .001) and endometrioid (p = .01) histologies when compared to clear cell histology. MGMT was significantly elevated in 93% of serous and endometrioid samples, compared to 62% of samples with clear cell histology. Those proteins most often underexpressed included Her2/neu, SPARC, and c-kit, seen in less than 1%, 4%, and 5% of specimens, respectively.

**Conclusions:**

PEP is a reliable and effective way of analyzing ovarian cancer specimens. PEP target identification does not appear to vary significantly with site evaluated, ovarian or other abdominal pelvic tissue, or primary versus recurrent disease. Variability in the expression of drug targets, including BCRP, ER, MGMT, and RRM1 could impact decision making pertaining to which therapeutic strategies carry the best chances for controlling disease.

**Electronic supplementary material:**

The online version of this article (doi:10.1186/2053-6844-1-4) contains supplementary material, which is available to authorized users.

## Background

Ovarian cancer is the fifth-leading cause of cancer death among women in the United States [1]. The current standard initial treatment of epithelial ovarian cancer includes surgical staging with optimal tumor debulking, followed by the administration of six cycles of intravenous chemotherapy with carboplatin and placlitaxel [2]. Although more than 80% of patients benefit from first-line therapy, tumor recurrence develops in nearly all patients, at a median of 15 months from completion of treatment [2]. Moreover, platinum resistance occurs in 25% of patients within 6 months from the last administration of platinum-agent [3], and the overall five-year survival rate of advanced stage disease is 37% [1]. For this reason, ovarian cancer is considered a systemic disease, and systemic therapies are being increasingly relied upon for treatment.

In the recurrent setting, tumor molecular profiling has been an area of recent investigation in an attempt to improve patient outcomes by employing targeted chemotherapeutic agents. In a pilot study, Von Hoff et al. [4] performed molecular profiling (MP) in 86 patients with refractory metastatic cancers. Of the 27% of patients who received targeted chemotherapy, the MP approach resulted in a longer progression-free survival than the regimen on which the patient had just experienced progression.

In another study, The Cancer Genome Atlas (TCGA) researchers reported DNA gene mutations on tumor samples in patients with high-grade serous ovarian carcinomas (HGS-OvCa) [3]. Their findings were significant for TP53 mutations being identified in nearly all tumors (96%), while BRCA1 and BRCA2 mutations were identified in 22% of tumors. Their results indicated that the mutational spectrum of HGS-OvCa is distinct from other histological subtypes of ovarian cancer. Further study would therefore warrant the investigation of targeted therapies based on the specific molecular alterations identified in a specific histology of ovarian cancer.

To date, there is modest data regarding MP of ovarian cancer specimens. Of the 86 patients in the Von Hoff study, only 5 had ovarian cancer [4]. Furthermore, the TCGA data was limited to specimens with high-grade serous histology. The objective of the current study, therefore, is to determine the protein expression profile of primary and recurrent ovarian cancer patients of all histological types in order to predict potential therapeutic targets for chemotherapy.

## Methods

The Target Now® (Caris Life Sciences®) database was accessed to obtain MP results for ovarian cancer tissue samples. De-identified results of Target Now®, as well as available histopathologic and clinical data from the Caris Life Sciences'® database were obtained after Institutional Review Board (IRB) approval. Because the research involved data collected previously for clinical utility (non-research purposes), this study qualified and was approved for expedited review by the IRB at St. Joseph’s Hospital and Medical Center. Data was transferred from Caris Life Sciences® to the investigators in the form of a limited data set in compliance with a Data Transfer Agreement.

### Immunohistochemistry

The database was used to analyze formalin fixed tissue samples for protein expression profile (PEP) by immunohistochemistry (IHC), which were obtained from women with primary and recurrent ovarian carcinoma, 62% and 37% respectively. Specimens were collected between January 2010 and December 2010. A total of 428 specimens were analyzed for 18 protein markers specific to the Target Now® PEP: Androgen Receptor, BCRP, c-kit, ER/PR, ERCC1, Her2/Neu, MGMT, MRP1, PDGFR, PGP, PTEN, RRM1, SPARC, TOP2A, TOPO1, TS, cMET, TUBB3. Analyses were performed by Caris Life Sciences® (Irving, Tx). The tests performed by Caris Life Sciences® were part of the Target Now® MP service. Staining protocols employed the Ventana Medical Systems, Inc. (Tucson, AZ) automated staining systems. Following heat-induced epitope retrieval, antibody incubation was for 20–40 minutes (antibody-specific), and visualization procedure was based on the staining system. Appropriate positive and negative control specimens and slides were included for all of the proteins tested. Slides were evaluated semi-quantitatively for staining intensity on a scale of 0 (no staining) to 3 and by the percentage of the tumor cells showing the reactivity. For IHC scoring appropriate positive and negative thresholds were defined and considered in the subsequent statistical analyses with intermediate and unknown results considered not evaluable. Comparison of IHC results with clinical variables was performed with Fisher’s exact test.

### Oligonucleotide microarray (MA) gene expression

A total of 67(25%) of the 264 primary specimens were analyzed for MA. Normal tissue from the abdominal pelvic cavity was used as a control organ. No specific site was ever designated as a control, the only requirement being that it was uninvolved with cancer. The arrays contain probes for 88 genes for which there is a therapeutic agent that could potentially interact with that gene. Those 88 genes are listed in Additional file 1: Table S1. The frozen tumor fragments for MA were placed in a glass tube on 0.5 mL of frozen 0.5 M guanidine isothiocyanate solution, thawed, and homogenized with a Covaris S2 (Covari, Woburn, MA). RNA was bound and then eluted. RNA was tested for integrity by assessing the ratio of 28S to 18S ribosomal RNA on an Agilent BioAnalyzer (Agilent, Santa Clara, CA). Tumor RNA (2 to 5 μg) and control RNA from a sample of a normal tissue representative of the tumor's tissue of origin (2 to 5 μg) were converted to cDNA and labeled during T7 polymerase amplification with contrasting fluor tagged (Cy3, Cy5) cytidine triphosphate. The labeled tumor and its tissue of origin reference were hybridized to an Agilent H1Av2 60-mer oligo array chip with 17,085 unique probes. The chips were then scanned on an Agilent Microarray Scanner (Agilent, Santa Clara, CA). Fluorescence intensity data were extracted, normalized, and analyzed using Agilent Feature Extraction Software. The MA was considered positive for a target if the difference in expression for a gene between tumor and control organ tissue was at a significance level of *P* ≤ .001. Cut points were chosen for gene expression of the cancer based on stringent *P* values (*P* < .001) compared with the normal mRNA expression levels. It was decided that using the mRNA level from the tissue of the organ of tumor origin would be the most informative comparison. To provide stringent quality control, these MA studies were performed in a Clinical Laboratory Improvement Amendments–certified environment.

### Statistical analysis

Generalized Linear Models (GLM) were used with binomial distribution and link logit specified to predict the dichotomous outcome variable overexpression from the categorical predictor histology. Histologies classified as other was set as the reference group for the computation of parameter estimate odds ratios. One GLM model per biomarker was conducted resulting in eighteen models. The False Discovery Rate ^(SITE)^ correction used to adjust for multiple comparisons for significant GLM models. SPSS version 20 was used for statistical analyses.

## Results

Specimens were obtained from 428 females with a median age of 62 years (Table 1). The majority of specimens analyzed by IHC were of serous and carcinoma histology (82%, Table 2). Thirty-five percent of specimens were poorly differentiated, while 27% were of unknown histological grade. Of the primary specimens, 62% were from adnexa, while 53% were obtained from a metastatic site and not the adnexa. Of the 67 specimens analyzed by MA, 55 (82%) were serous histology, 4 (6%) endometrioid, 2 (3%) clear cell, and 6 (9%) other. An advanced-stage diagnosis was represented in 60% of specimens. Five of the eighteen binomial GLM models predicting protein overexpression were significant. Significant models were for BCRP, ER, MGMT, RRM1, and PTEN at the trend level (Table 3). Twelve pairwise post hoc comparisons were performed for each significant model resulting in sixty post hoc contrasts (Table 4).

**Table 1:** **Patient characteristics at diagnosis**

	(N)	Percent%
**TOTAL**	428	
**Median age (years)**	61.7	
**Tumor histology**		
Serous	351	82
Clear cell	21	5
Endometrioid	28	7
Other	28	6
**Tumor stage**		
I	36	8
II	24	6
III	224	52
IV	34	8
Missing	110	26
**Tumor grade**		
Well differentiated	117	27
Moderately differentiated	43	10
Poorly differentiated	151	35
Unknown	117	28
**Timing**		
Primary	264	62
Recurrent	157	37
Unknown	7	1

**Table 2:** **Counts and proportions of overexpression for biomarkers by histology**

Biomarker	Serous mean (sd)	Clear cell mean (sd)	Endometrioid mean (sd)	Other mean (sd)
1 Androgen receptor	.27(.44)	.10(.31)	.27(.45)	.12(.33)
2 BCRP*	.85(.36)	.85(.37)	.65(.49)	.72(.46)
3 c-kit	.05(.22)	.00(.00)	.00(.00)	.00(.00)
4 ER*	.47(.50)	.14(.36)	.50(.51)	.39(.50)
5 ERCC1	.24(.43)	.33(.48)	.04(.19)	.25(.44)
6 Her2/Neu	.01(.09)	.00(.00)	.00(.00)	.00(.00)
7 MGMT***	.93(.25)	.62(.50)	.93(.26)	.89(.32)
8 MRP1	.89(.32)	.85(.37)	.92(.27)	.92(.27)
9 PDGFR	.18(.38)	.15(.37)	.23(.43)	.16(.37)
10 PGP	.19(.39)	.24(.47)	.21(.42)	.14(.36)
11 PR	.39(.49)	.19(.40)	.50(.51)	.36(.49)
12 PTEN	.65(.48)	.48(.51)	.43(.50)	.61(.50)
13 RRM1**	.47(.50)	.14(.36)	.24(.44)	.57(.50)
14 SPARC MONO	.11(.32)	.24(.47)	.10(.30)	.16(.37)
15 SPARC POLY	.04(.20)	.00(.00)	.00(.00)	.00(.00)
16 TOP2A	.39(.49)	.20(.41)	.23(.43)	.40(.50)
17 TOPO1	.89(.32)	.86(.36)	.89(.32)	.93(.26)
18 TS	.15(.35)	.05(.22)	.08(.27)	.16(.37)

*P < .05, **P < .01, ***P < .001.

**Table 3:** **Parameter estimates predicting overexpression from histology**

		OR	95% CI	***P***	Wald chi-square
BCRP	Carcinoma and serous	2.21	.88-5.58	0.09	8.39, p = .039
	Clear cell	2.2	.49-9.94	0.3	
	Endometriod	0.735	.22-2.41	0.61	
ER	Carcinoma and serous	1.39	.63-3.05	0.42	7.76, p = .051
	Clear Cell	0.26	.06-1.09	0.07	
	Endometriod	1.55	.54-4.46	0.42	
MGMT	Carcinoma and serous	1.64	.46-5.81	0.48	18.57, p < .001
	Clear cell	0.2	.04-.86	0.03	
	Endometriod	1.56	.24-10.14	0.64	
PTEN	Carcinoma and serous	1.19	.54-2.61	0.67	7.01, p = .071
	Clear cell	0.59	.19-1.85	0.36	
	Endometriod	0.49	.17-1.41	0.18	
RRM1	Carcinoma and serous	0.67	.31-1.46	0.32	13.06, p = .005
	Clear cell	0.13	.03-.52	0.004	
	Endometriod	0.25	.08-.78	0.017

**Table 4:** **Post hoc comparisons for significant glm models predicting overexpression**

		BCRP					ER				
		Mean diff	SE	95% LCL	95% UCL	p	Mean diff	SE	95% LCL	95% UCL	p
Carcinoma and serous	Clear cell	0	0.08	-0.16	0.16	.993	0.33	0.08	0.17	0.49	.000*
Carcinoma and serous	Endometriod	0.2	0.10	0.01	0.38	.039	-0.03	0.10	-0.22	0.17	.783
Carcinoma and serous	Other	0.13	0.09	-0.05	0.31	.155	0.08	0.10	-0.11	0.27	.405
Clear cell	Endometriod	0.2	0.12	-0.04	0.44	.110	-0.36	0.12	-0.60	-0.12	.003*
Clear cell	Other	0.13	0.12	-0.11	0.37	.279	-0.25	0.12	-0.48	-0.02	.037
Endometriod	Other	-0.07	0.13	-0.32	0.19	.609	0.11	0.13	-0.15	0.37	.417
		**MGMT**					**RRM1**				
		**Mean diff**	**SE**	**95% LCL**	**95% UCL**	**p**	**Mean diff**	**SE**	**95% LCL**	**95% UCL**	**p**
Carcinoma and serous	Clear cell	0.31	0.11	0.10	0.52	0.00*	0.33	.081	.17	.49	.000*
Carcinoma and serous	Endometriod	0.00	0.05	-0.10	0.10	0.95	0.22	.086	.05	.39	.010*
Carcinoma and serous	Other	0.04	0.06	-0.08	0.16	0.52	-.10	.097	-.29	.09	.311
Clear cell	Endometriod	-0.31	0.12	-0.54	-0.08	0.00*	-.11	.112	-.33	.11	.338
Clear cell	Other	-0.27	0.12	-0.51	-0.04	0.02	-0.43	.121	-.67	-.19	.000*
Endometriod	Other	0.04	0.08	-0.11	0.18	0.95	-0.32	.124	-.56	-.08	.010*
		**PTEN**									
		**Mean diff**	**SE**	**95% LCL**	**95% UCL**	**p**					
Carcinoma and serous	Clear cell	.17	.112	-.05	.39	.128					
Carcinoma and serous	Endometriod	0.22	.097	.03	.41	.024					
Carcinoma and serous	Other	.04	.096	-.15	.23	.679					
Clear cell	Endometriod	.05	.144	-.23	.33	.740					
Clear cell	Other	-.13	.143	-.41	.15	.359					
Endometriod	Other	-.18	.131	-.44	.08	.174					

*P < 0.05.

ER protein was significantly overexpressed in 47% of serous and 50% of endometrioid samples, while only elevated in only 14% of clear cell samples. Post-hoc testing demonstrated the ER protein was significantly overexpressed in serous (p < .001) and endometrioid (p = .003) histologies when compared to clear cell histology (Table 2). The MGMT and RRM1 biomarkers were also significantly overexpressed in serous (p < .001) and endometrioid (p = .01) histologies when compared to clear cell histology. MGMT was significantly elevated in 93% of serous and endometrioid samples, compared to 62% of samples with clear cell histology. Only 14% of clear cell samples were found to have overexpression of RRM1, compared to 47% of serous and 25% of endometrioid samples. Those proteins most often underexpressed included Her2/neu, SPARC, and c-kit, seen in less than 1%, 4%, and 5% of specimens, respectively. None of the post hoc comparisons for histology were significant for biomarkers BCRP and PTEN with the FDR correction applied.

**Figure 1:**
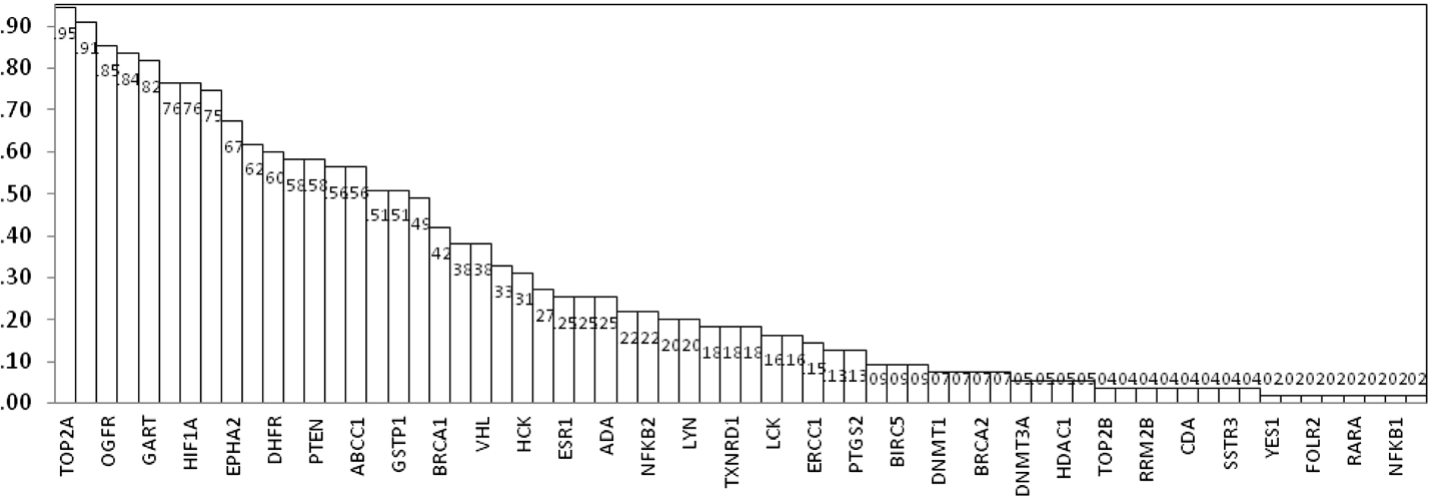
**Proportion of protein overexpression by gene for serous ovarian cancer.** The figure reports the fraction of serous ovarian cancer samples with overexpressed mRNA by gene. There was no differential expression of genes with regard to stage, grade, or race.

There were 88 genes evaluated by MA gene expression and included in statistical analysis for association with histology. Those genes most overexpressed, defined as a difference in expression of mRNA between tumor and control organ tissue at a significance level of *P* ≤ .001, in serous histology included TOP2A, MSH2, OGFR, RRM2, GART, and PARP1. A description of proportion of positive expression is shown in Figure 1. There were fourteen genes that were never expressed in serous histology and are not shown in Figure 1: ABCG2, AR, CES2, KIT, MS4A1, PDGFRA, PDGFRB, POLA1, RXRB, SPARC, SSTR1, SSTR2, SSTR4, and TOP1. There was no difference in protein or gene overexpression identified when analyzed by age, FIGO stage, grade, primary or recurrent tumor, and ovary or other biopsy site.

## Discussion

TCGA project has analyzed post-transcriptional messenger RNA expression, microRNA expression, promoter methylation, and DNA copy number in 489 high-grade serous ovarian adenocarcinomas [3]. They reported that high-grade serous ovarian cancer is characterized by TP53 mutations in almost all tumors (96%); and a low prevalence but statistically significant recurrent somatic mutations in nine further genes including NF1, BRCA1, BRCA2, RB1, and CDK12. Pathway analyses suggested that homologous recombination was a potentially important pathway and was defective in about half of the high grade serous cancers analyzed. NOTCH and FOXM1 signaling were involved in serous ovarian cancer pathophysiology.

Molecular PEP is a powerful approach to identify clinical markers for diagnosis and prognosis as in epithelial ovarian cancer. In a previous smaller study, a tissue array composed of 244 serous tumors of different grades (0–3) and stages (I–IV) was evaluated by comprehensive IHC for proteins not necessarily associated with response to selective chemotherapeutic agents [5]. Ccne1, Ran, Cdc20, and Cks1 showed significant differences of expression in association with the clinical stage of disease. The application of these biomarkers in both the initial diagnosis and prognostic attributes of patients with epithelial ovarian tumors could prove to be useful in patient management.

The main objective of this study was to characterize and identify the characteristics of epithelial ovarian cancers through PEP of ovarian tumors in order to predict targeted therapies. The current study is one of the largest PEP analyses of epithelial ovarian cancers in the literature. This study demonstrates that PEP is a reliable and effective way of analyzing ovarian cancer specimens. In this study, protein expression target identification did not appear to vary significantly with the site evaluated, ovarian or other abdominal pelvic tissue, or with primary versus recurrent disease.

Several markers have been identified which may predict a specific tumor’s response to chemotherapy (Table 3). Breast cancer resistance protein (BCRP), an atypical drug efflux pump, mediates multidrug resistance in breast cancer, as well as other cancer types, by reducing the intracellular concentration of cytotoxic drugs [6]. BCRP has been described in breast, colon, gastric cancer, and fibrosarcoma cell lines but has also had documented overexpression in ovarian cancer cell lines [7]. Studies in topotecan-resistant ovarian cancer cell lines have demonstrated a substantial overexpression of BCRP. Moreover, these cell lines demonstrated resistance to other topoisomerase I inhibitors, SN-38 (the active metabolite of irinotecan, and 9-aminocamptothecin, as well as the topoisomerase II inhibitor, mitoxantrone [6, 7]). Topotecan, etoposide, mitoxantrone, 5-FU, anthracyclines such as doxorubicin and pirarubicin, as well as methotrexate have all been identified as substrates of BCRP [8, 9], and therefore would be less effective in tumors that overexpress this protein. However, paclitaxel, vincristine, vindesine, mitomycin c, and cisplatin are not mediated by BCRP [9] and would likely be more successful in treating patients with tumors that overexpress BCRP.

Tumors which overexpress estrogen receptor (ER) may have an enhanced response to anti-estrogens such as megestrol, tamoxifen, and aromatase inhibitors. These therapies have been used extensively in the treatment of ER-positive breast cancer. However, there is sparse data on ER-positivity in ovarian tumors. In the ‘MALOVA’ Ovarian Cancer Study, the investigators identified that ER was expressed in 36% of epithelial ovarian tumors [10]. The authors suggested ER is a prognostic indicator of improved survival in ovarian cancer patients, though no targeted therapy was used in this study. Data on the use of anti-estrogens in the treatment of ovarian cancer is limited to phase II trials with results indicating these therapies, if successful, are predominantly able to achieve disease stabilization, with only marginal rates of partial or complete treatment responses [11–15].

MGMT, or O6-methlyguanine-DNA methyltransferase, is an enzyme that repairs methylated DNA and plays a crucial role in the protection against alkylating agents [16, 17]. MGMT expression in cancer cells can inhibit the success of chemotherapy treatment with alkylating agents, which work by triggering DNA methylation [18]. If MGMT is overexpressed in the tumor, alkylating agents such as temozolomide may be less effective. Low expression of MGMT has been associated with successful response to treatment with temozolomide [19]. Furthermore, inhibition of MGMT activity has been shown to increase the toxicity of alkylating agents [17, 18].

RRM1 is a subunit of Ribonucleotide reductase (RR), an enzyme that acts as the rate-limiting step in DNA synthesis, as it is the only known enzyme to convert ribonucleotides to deoxyribonucleotides for DNA polymerization and repair. It functions with the p53-regulated RRM2 homologue p53R2 and is important in DNA repair secondary to genotoxic stress [20]. Gemcitabine is an analog of deoxycytidine and is converted intracellularly into active diphosphate and triphosphate nucleosides, which become incorporated into the DNA chain, leading to termination of chain elongation and inhibition of DNA synthesis [21]. In preclinical studies, increased RRM1 expression and activity have been shown to be markers for gemcitabine resistance, suggesting that RRM1 expression is a negative predictor of gemcitabine efficacy [20–24]. However, patients with low as compared to high levels of tumoral RRM1 expression had improved survival when treated with gemcitabine-based therapy [25].

In this particular study, BCRP, ER, MGMT, and RRM1 proteins were overexpressed in 85%, 47%, 93%, and 47% of serous carcinomas, respectively. As these proteins have been shown to be associated with various resistances to specific chemotherapeutic agents, targeted therapy may be helpful in identifying successful treatments. Because of the increased expression of BCRP, MGMT, and RRM1 identified in serous carcinomas, in theory chemotherapeutic agents such as etoposide, adriamycin, temozolomide, and gemcitabine may be avoided in the treatment of serous tumors that overexpress these biomarkers and considered only for other histologies of ovarian cancer.

On the other hand, for a segment of the ovarian cancer population, drugs that have not been developed in ovarian cancer, such as temozolomide, could be useful for patients with the right biomarker profile, such as low MGMT expression, perhaps even in preference to giving a standard drug like topotecan to a patient with suboptimal biomarker expression, such as low TOPO1. In this way, PEP offers a laboratory-based approach to drug selection, rather than the empiric method of selecting drugs blindly. Accordingly, PEP could impact medical decision-making by prioritizing which standard agents are most likely and least likely to offer benefit to the individual patient, as well as identifying drugs outside the standard armamentarium with previously unrecognized potential to control disease. Admittedly, future studies measuring outcomes in patients undergoing PEP are sorely needed before PEP becomes a standard of care.

This study is limited by its retrospective design and descriptive nature, and the absence of clinical information such as response rates, disease status, which might allow correlation with response at this time. Another limitation of the current study pertains to the absent inclusion of a variety of molecular markers that appear to play a significant role in driving malignant behavior or in producing drug resistance. These include P53 and PIK3CA mutation, cMET, NOTCH, and FOXM1. To the extent that pathways that cause chemotherapeutic resistance were not assayed, the report of optimal protein expression for a given patient could over-estimate the chance for benefit from the associated agent.

## Conclusion

We observed that a majority of patients with serous histology demonstrated protein expression associated with resistance to one or more chemotherapeutic agents. Furthermore, the resistance pattern in each individual cancer was unpredictable. Future studies could include clinical benefit analysis of MP in ovarian cancer patients in prospective trials or analysis of MA gene expression in ovarian tumors. Given the high proportion of advanced stage at diagnosis and the proclivity of ovarian cancer recurrence, efforts such as MP have the potential to make a large impact on survival of this disease.

## Electronic supplementary material

Additional file 1: **Pairing of Protein Expression Profile (PEP) with Agents.** (DOCX 35 KB)

Below are the links to the authors’ original submitted files for images.

Authors’ original file for figure 1
