# Supplementary material for: Protein profiling of ovarian cancers by immunohistochemistry to identify potential target pathways
Source: Gynecol Oncol Res Pract. 2014 Sep 30;1:4. doi: 10.1186/2053-6844-1-4 (PMC4877732; doi:10.1186/2053-6844-1-4)
Supplement: Supplementary file 1 — Additional file 1: Pairing of Protein Expression Profile (PEP) with Agents. (DOCX 35 KB) [file 40661_2014_3_MOESM1_ESM.docx]

Table A1.

Pairings of Targets and Drugs

| **Potential Target** | **Agents Suggested as Interacting With the Target** |
| --- | --- |
| IHC |  |
| EGFR | Cetuximab, erlotinib, gefitinib |
| SPARC | Nanoparticle albumin-bound paclitaxel |
| c-KIT | Imatinib, sunitinib, sorafenib |
| ER | Tamoxifen, aromatase inhibitors, toremifene, progestational agent |
| PR | Progestational agents, tamoxifen, aromatase inhibitor, goserelin |
| Androgen receptor | Flutamide, abarelix, bicalutamide, leuprolide, goserelin |
| PGP | Avoid natural products, doxorubicin, etoposide, docetaxel, vinorelbine |
| HER2/NEU | Trastuzumab |
| PDGFR | Sunitinib, imatinib, sorafenib |
| CD52 | Alemtuzumab |
| CD25 | Denileukin diftitox |
| HSP90 | Geldanamycin, CNF2024 |
| TOP2A | Doxorubicin, epirubicin, etoposide |
| Microarray |  |
| *ADA* | Pentostatin, cytarabine |
| *AR* | Flutamide, abarelix, bicalutamide, leuprolide, goserelin |
| *ASNA* | Asparaginase |
| *BCL2* | Oblimersen sodium |
| *BRCA2* | Mitomycin |
| *CD33* | Gemtuzumab ozogamicin |
| *CDW52* | Alemtuzumab |
| *CES-2* | Irinotecan |
| *DCK* | Gemcitabine |
| *DNMT1* | Azacitidine, decitabine |
| *EGFR* | Cetuximab, erlotinib, gefitinib |
| *ERBB2* | Trastuzumab |
| *ERCC1* | Cisplatin, carboplatin, oxaliplatin |
| *ESR1* | Tamoxifen, aromatase inhibitors, toremifene, progestational agent |
| *FOLR2* | Methotrexate, pemetrexed |
| *GART* | Pemetrexed |
| *GSTP1* | Platinum |
| *HDAC1* | Vorinostat |
| *HIF1α* | Bevacizumab, sunitinib, sorafenib |
| *HSPCA* | Geldanamycin, CNF2024 |
| *IL2RA* | Aldesleukin |
| *KIT* | Imatinib, sunitinib, sorafenib |
| *MLH-1* | Gemcitabine, oxaliplatin |
| *MSH1* | Gemcitabine |
| *MSH2* | Gemcitabine, oxaliplatin |
| *NFKB2* | Bortezomib |
| *NFKB1* | Bortezomib |
| *OGFR* | Opioid growth factor |
| *PDGFC* | Sunitinib, imatinib, sorafenib |
| *PDGFRA* | Sunitinib, imatinib, sorafenib |
| *PDGFRB* | Sunitinib, imatinib, sorafenib |
| *PGR* | Progestational agents, tamoxifen, aromatase inhibitors, goserelin |
| *POLA* | Cytarabine |
| *PTEN* | Rapamycin (if low) |
| *PTGS2* | Celecoxib |
| *RAF1* | Sorafenib |
| *RARA* | Bexarotene, all-*trans*-retinoic acid |
| *RXRB* | Bexarotene |
| *SPARC* | Nanoparticle albumin-bound paclitaxel |
| *SSTR1* | Octreotide |
| *TK1* | Capecitabine |
| *TNF* | Infliximab |
| *TOP1* | Irinotecan, topotecan |
| *TOP2A* | Doxorubicin, etoposide, mitoxantrone |
| *TOP2B* | Doxorubicin, etoposide, mitoxantrone |
| *TXNRD1* | Px12 |
| *TYMS* | Fluorouracil, capecitabine |
| *VDR* | Calcitriol |
| *VEGF* | Bevacizumab, sunitinib, sorafenib |
| *VHL* | Bevacizumab, sunitinib, sorafenib |
| *ZAP70* | Geldanamycin, CNF2024 |
