# Supplementary figures and images for: Protein profiling of ovarian cancers by immunohistochemistry to identify potential target pathways
Source: Gynecol Oncol Res Pract. 2014 Sep 30;1:4. doi: 10.1186/2053-6844-1-4 (PMC4877732; doi:10.1186/2053-6844-1-4)

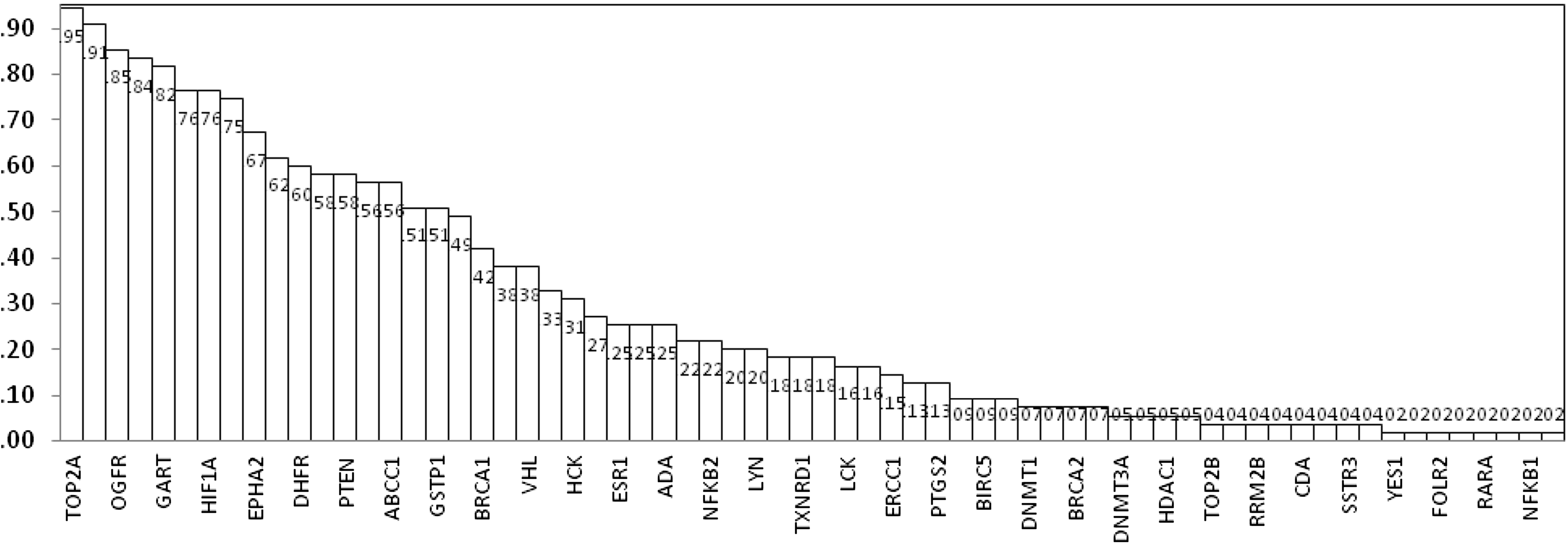

Supplement: Supplementary file 2 — Authors’ original file for figure 1 [file 40661_2014_3_MOESM2_ESM.tiff]
